# Exploring social innovation for transforming food systems

**DOI:** 10.1098/rstb.2024.0156

**Published:** 2025-09-18

**Authors:** Fergus Lyon, Bob Doherty, Katerina Psarikidou, Ian Vickers, Alise Kirtley

**Affiliations:** ^1^ Centre for Enterprise, Environment and Development Research (CEEDR), Middlesex University London, London NW4 4BT, UK; ^2^ School for Business and Society, University of York, York YO10 5DD, UK; ^3^ Science Policy Research Unit, University of Sussex, Brighton BN1 9RH, UK; ^4^ Institute of Development Studies, University of Sussex, Brighton BN1 9RE, UK

**Keywords:** social innovation, food systems, transformation, behaviour change, business model, community engagement

## Abstract

To achieve transformation in food systems, there is a need for social change and innovation in how people grow, trade and consume food. This paper explores how the concept of social innovation can help understand the processes of developing novel solutions that potentially contribute to transforming food systems for health and sustainability. Drawing on a set of case studies in the UK, our analysis shows that food system social innovation is found in: place-based initiatives such as community hubs, cafes and therapeutic growing space; supply chains and food access for social justice and sustainability; and food behaviour change activities that alter consumption and purchasing. We also identify key social innovation processes, including collaborative partnerships for co-learning and developing solutions, developing alternative social enterprise business models and engaging with policy and institutional change. Challenges facing social innovation are identified, along with processes for scaling up impact on food systems.

This article is part of the theme issue ‘Transforming terrestrial food systems for human and planetary health’.

## 1. Introduction

Mounting evidence of the harmful effects of the dominant system of food provisioning has prompted calls for fresh thinking to overcome the ‘false economy of Big Food’ [1] and related shortcomings of UK public policy towards food and health [2–4]. Transforming food systems for health and sustainability requires social innovation, defined as the new strategies, practices, organizational designs and collaborations that address unmet social needs [5]. As a multi-stakeholder process and mode of governance, SI aims to be more inclusive, participatory and attuned to social well-being compared with innovation that is primarily motivated by private profit and technological development.

This paper examines the concept of social innovation, its practice and potential by empirically examining UK cases of social innovation addressing food issues facing consumers, supply chains and producers. We show how the concept of social innovation allows a richer understanding of food system transformation—defined by Buckton *et al*. [6] as going beyond changing technologies, policies and behaviours, to include change in underlying structures, power relations, beliefs, values and worldviews (see also [7]).

Existing research has explored the meaning and processes of social innovation [8,9]. The aim of this paper is to develop a framework for understanding the dimensions and processes of food systems social innovation, the barriers faced by social entrepreneurs and organizations working in food systems, and the ways they address these barriers in order to sustain and scale their initiatives.

The paper starts with an overview of the concept of social innovation drawn from the literature, from which we derive our research questions, followed by a presentation of the methodology and analysis of seven case studies of social innovation. Our discussion and conclusions draw out some wider implications for food-related policy, practice and collaborative/transdisciplinary research.

## 2. Literature review

Although the concept of social innovation has a long history [10], it has gained considerable interest within academia and traction with policy makers over the last decade [11,12]. Social innovation is used to describe the development of new strategies, concepts and organizations in response to social needs in areas such as health, education, community development, strengthening civil society, and environmental sustainability [5]. This also includes new social relationships and collaborations that are ‘designed by and for society’ and contribute to improved well-being [11]. In this paper, we focus on those practices in the food system that have social and environmental aims prioritized at all stages to enact incremental change as well as a diversity of intentions for food system transformation with regards to structures, power relations, beliefs, mindsets and worldviews that need to change [7]. This is distinct from other forms of technology- and profit-focused innovation from which social implications may unintentionally emanate [13].

Recent scholarly attention results in part from discontent with the mainstream understanding of innovation as largely involving the development of new products and processes that are market-led and technology-based [12,14–16]. However, it is important to note the conceptual ambiguity and contested nature of social innovation definitions [9]. For instance, the definition of social innovation adopted by the European Union (EU) has been criticized for being too broad and for including less transformational activities that do not adequately interrogate underlying power dimensions [17]. There are also debates over whose voices are heard in the development of social innovations [18].

With regard to the transformational potential of innovations, it is important to consider the degree of novelty of different types of innovation. Although some innovations may be completely new to the world, many are best understood as more incremental and novel in relation to the specific contexts (sector, place, organization) of their introduction [19].

Research on social innovation has used the term to describe the product/services developed and the processes, governance or business models that have potential to contribute to systemic and transformative change. In relation to FS, the products and services may relate to forms of food production that address well-being of growers, care for the environment and benefits for consumers [12]. There is also research that explores ways of addressing food poverty, shortening food supply chains and encouraging local production [18,20,21].

In terms of social innovation as a mode of governance [22], there is research on multi-stakeholder partnerships and networks [23] to help orchestrate systemic change, including by linking small-scale enterprises and community initiatives to larger organizations, policy and regulations [24]. At the local and regional economy level, for instance, producer food hubs act as intermediaries to bring together small producers [25,26].

It is also important to examine the process of social innovation itself with greater attention being paid to socially innovative business models with democratic governance [13,27] and engagement models that are needed to enact systemic change. These collaborative partnerships can be part of the social innovation process, ensuring a wide range of stakeholders are involved in developing and scaling up social innovations. In food studies, there is a long tradition of encouraging such participation in agri-food research [28,29]. This leads to the following research question: *What are the dimensions and processes of social innovation practice with intent for transforming food systems?*

These approaches are not without their challenges in terms of ensuring participation from stakeholders and ensuring the more disadvantaged groups are not excluded. Smith & Raven [30] highlight the path-dependent nature of institutional development, the power relations and constraining influence of incumbent interests, and the limited powers of ‘green niche challengers’ to effect change (see also [31]). Relatedly, Purna & De Paoli [9] point to the risk of social innovation's meaning and usage—i.e. as a malleable concept open to varied interpretations—being appropriated, resisted and diverted away from its common good intentions by powerful mainstream actors and vested interests (see also [32–34]).

Further challenges come from the lack of resources and funding for innovation and the lack of an enabling food policy environment and governance framework to encourage change [35]. The state is a key strategic actor through its public policy and decision-making related to financial investment, innovation and enterprise support, regulation and standard setting, and procurement rules. Parsons & Barling [36] usefully map the policy toolbox for food systems transformation and provide a taxonomy of the types of policy levers available to government and its agencies. However, government responses can be piecemeal and lack ambition [3]. This leads to the following research question: *What are the challenges to overcome in developing these innovations?*

Scaling-up is key for social innovations to overcome current challenges. Although some social innovations may remain as small-scale niche practices, scaling-up, diffusion and ‘beyond niche’ development can include the growth of the innovating enterprise itself (i.e. in terms of turnover, employment size and beneficial impacts) and the replication and adaptation of concepts and organizational models in new contexts. Social innovation can also include open knowledge sharing to influence the practices of other enterprises and of policy makers at local/regional, national and international levels [30,35,37].

Scaling also requires a supportive environment and networks, or what Pel *et al*. [22] call a supportive social innovation ecosystem. These processes of social innovation can be supported by being embedded locally, nationally and internationally in the design of research and innovation programmes of academia, civil society organizations and the public sector. This can include the transdisciplinary capabilities that bring together different disciplines, professions and perspectives [38]. Our third research question is therefore: *How can social innovations be amplified and lead to future transformations?*

## 3. Methods

Our paper draws on in-depth qualitative cases of seven organizations, all of which have the intent to change the food system and are purposely selected to capture the diversity of social innovation practices and processes (see table 1). Drawing on recent conceptualizations of food system transformation and its systemic character [7], our qualitative cases cover all interconnected elements of the system from farm to fork. These cases were drawn from a wider sample frame of social innovations identified as part of a review of social innovation within the Transforming UK Food Systems Programme [24], which led to the selection of cases from three of the funded research projects that had a particular focus on social innovation and hybrid business models. As also summarised in the supplementary material provided , data include multiple/mixed methods (interviews, focus groups, observation and secondary data) to triangulate the findings with an emphasis on in-depth interviews with social entrepreneurs/organization leaders, staff and user community stakeholders.

**Table 1.** Case studies of social innovation.

organization/location	main service area + food-related activity	year established	no. staff + volunteers	legal form
Cultivate (Powys)	local growing, food hub, school/community projects and social prescribing	2014	10 20	community benefit society, company limited by shares
London Early Years Foundation	children’s services + Nursery Chef initiative	incorporated 1988 (but dates back to 1903)	814 0	registered charity, private limited company by guarantee
Windmill Hill City Farm (Bristol)	community hub and health/well-being + local/organic food	1976	124 300	charity and company limited by guarantee
Social Adventures (Salford)	community health and well-being + therapeutic growing, food hub	2011	50 30	community benefit society
Brighton and Hove Food Partnership	coordinating organizations and actions to fix the food system; community research; Brighton and Hove city food strategy; community kitchen; land use project	2003	20 150	non-profit organization
Organic Pantry (Tadcaster, Yorkshire)	integrated regenerative organic fruit and vegetable farmer with organic box scheme in Leeds and York; produce into school contracts	2015	24	private limited company with share capital
Yorkshire Grain Alliance	network of bakers, millers, traders, researchers and community members; heritage varieties of grain and regenerative agriculture	2020	25 members	network of stakeholders

The transcripts of interviews and other data were analysed in a three-stage process. In the first stage, key issues related to social innovation were identified and an initial coding scheme developed. In the second stage, this coding scheme was combined with insights from the literature and the results of a review of social innovation across the Transforming UK Food Systems research programme [24]. This identified codes related to areas of social innovation practice, challenges faced, and approaches to scaling social innovation in each case study. In the third stage of analysis, the coded material was analysed further to draw out the key themes of food system social innovation, specific challenges and the approaches to scaling. This then led to the development of the framework for understanding social innovation in food systems.

## 4. Findings of the case studies

### (a) Dimensions of social innovation in the food system

#### (i) Collaborative place-based services

Many of the projects take a place-based collaborative approach with a focus on a particular locality, city or region. Examples include community cafés or community kitchens which can act as community hubs, like in the case of Cultivate. Windmill Hill City Farm (WHCF) links its café to the community growing space and uses this to bring groups, such as supper clubs, together, and start conversations about healthy food: ‘such a great way of getting rid of barriers in all sorts of ways, whether that’s cultural or in terms of people’s mental styles or whatever’ (WH2)^1^ . Both Cultivate and WHCF have been supporting local growers, by hiring out processing, bottling and labelling equipment.

The Social Adventures (SA) mental health and well-being programmes use the community café as a Welcome Hub, providing a hot, free meal every lunchtime. They designed the kitchen as ‘open plan and visible from the dining tables, which facilitates a connection between the cooks and the diners and establishes a social setting’ (SA4). In Brighton and Hove, the community kitchen provides both private and community classes in response to emerging community needs. As explained, ‘when the energy crisis hit, we started developing low energy cookery classes, and those were for people on low incomes’ (BH1).

With regard to food growing as a therapeutic activity, SA*,* for example, took over a closed retail garden centre and used part of the site for the food club and community garden, aiming to tackle mental health issues. At times these were linked to ‘social prescribing’ activities, where organizations obtain public funding for delivering therapeutic horticulture. Cultivate used this funding to develop other services to bring people onto the land and ‘design the gardens with the residents in mind, offering a calm and colourful space for people to relax and enjoy’ (C1).

#### (ii) Food access and supply chains

Social innovations are key in introducing new structures, relations and values around food supply practices that also address power imbalances. They aim to encourage access to local, affordable and sustainable food, like in the case of the Brighton and Hove Food Partnership (BHFP), who aspire to shorten the supply chain by ‘engag[ing] food producers, the council and different people to try and bring a lot of that food that they're producing into the city rather than diverted to international markets’ (BHFP2).

Supply chain social innovations are also found in relation to public sector food procurement being used to provide sustainable and healthy food. Organic Pantry (OP) won two large contracts to supply 500 schools with prepacked quartered regeneratively grown potatoes preserved in lemon juice. Cultivate has also been working on developing local food for schools in Powys, Wales, and London Early Years Foundation (LEYF) has been trying to source more locally for their nurseries but have faced challenges from the incumbent suppliers controlling the supply chain and not wanting to change behaviour.

These supply chain social innovations, such as the OP local vegetable box scheme, can be a driver for transitions to more sustainable farming practices. Running a box scheme requires strong collaboration with other growers, with reciprocal supply relationships to manage seasonality and ensure a variety of produce in each weekly box. Building on their direct links to consumer markets offering them a premium for their products, they are able to invest in agroforestry and novel grazing management and use this as part of their Future Farmers Network, sharing good practice on regenerative farming.

The Yorkshire Grain Alliance Network (YGAN) is another social innovation-supporting local, shorter and socially just supply chains by linking farmers to both flour mills and independent bakers. It aims to support sustainable farming practices, with the relationships and proximity allowing transparency and setting of fair prices for farmers and millers.

Social innovations are also designed to contribute to affordability of good food. Cultivate has been involved in using surplus food to create ready meals and has planted fruit trees and other produce within community spaces. Food poverty is an issue for most of the cases, with SA taking the innovative step to develop a food bank into a ‘people’s supermarket’ to give more choice. These approaches are all examples of changing values, structures and power relations with potential to transform the food system.

#### (iii) Education and behaviour change

There are a range of food social innovations related to education and activities that seek to have a positive influence on peoples’ dietary habits and upon wider patterns of consumption and production. Social innovations leverage their relationships and novel ways of engaging with people, creating the potential to change people’s beliefs and values around food. In this way, the SIs can be contrasted with the ‘top-down’ traditional activities of public health programmes, which have faced challenges in changing food consumption-related behaviour.

For example, LEYF realized that traditional ‘preaching’ approaches to parents were not working, and it wanted novel ways to help children model healthy food behaviour and have knowledge about food, and to encourage sustainability in nursery food. Its Nursery Chef programme aimed to change the role of those preparing food so they became integral parts of the education activities where children could learn about healthy and sustainable food.

The analysis of the cases in this paper shows other examples of working with young people through schools. In four of the cases, the social innovation included encouraging school visits, with WHCF hosting over 2500 children per year. OP hosts school tours to show organic and regenerative practice, and YGA sets up school visits to flour mills. They (YGA) also regularly organize community workshops in disadvantaged areas on baking and regenerative farming, while BHFP runs school cookery classes as part of its community kitchen programme. Work with young people does not necessarily have to be through schools, and Cultivate has been developing ways to engage with people on healthy eating by designing a ‘Top Trumps’ card game educating people about different foods.

Social innovations based on community learning found in five of the cases. SA and Cultivate use food as a way to engage with people for wider well-being activities, and develop cookery classes and horticulture training for community members. WHCF runs a programme that puts carbon footprint details on its café menu, as a way to influence the behaviour of customers. LEYF also uses its Nursery Chef programme to influence food consumption behaviour at home. It works with parents by sending food and recipe cards as ‘homework’ requiring children and parents to work together. Similarly, WHCF has been working with parents, grandparents and carers of nursery children as a way of engaging and educating the wider community about food.

Behaviour change and education are also found in social innovations related to training members of the community, practitioners, staff and partners in collaborating organizations. The BHFP established a programme for training community researchers to undertake research around food with local communities: ‘we, as a team, it’s given us a chance to collaborate to work together, to develop new skills … We've each found our sort of niche … in terms of co-writing, copy or editing’ (BHFP2). Similarly, LEYF has set up the Nursery Chef Training Academy, allowing it to scale up the impact by spreading the initiative across the chain of 39 nurseries and offering training to chefs from other organizations. A key challenge it faces is finding ways to cover the cost of taking people out of work for this training.

### (b) Processes of social innovation in food systems

#### (i) Collaborative partnerships for co-creation

In our analysis, social innovation can also be seen as a process allowing solutions to be identified and addressed. A key element found in all the cases was the need for participatory approaches where neglected voices can be brought in, with the intention to shift existing dynamics and power relations for food systems transformation. This was challenging in some contexts, with Cultivate finding that working with existing community engagement activity can help access those most in need, but still some parts of the community were at risk of being excluded. WHCF found innovative ways of using artists to help engagement: *‘*Last year we had an artist come in … and they just collar people as they came through and ask them to add to this huge drawing that he’d sketched up. It was really lovely of what we all think about the farm’ (WH1).

The community researchers of BHFP are an example of both an innovation in themselves and playing a bridging role to harness other social innovations. ‘Our main role is being a part of the community, so that we are kind of a bridge … They can talk to us about things and give us their ideas and experiences really about all different types of food, how they eat it, how they find it, their fears about it, hopes about it. And so that they can make real policy change hopefully with u*s*’ (BHFP1).

Collaborative partnerships between the social innovating organization and other civil society organizations were identified in many of the cases. For example, LEYF was collaborating with an organization that reduces waste by collecting food near its expiry dates and giving it to local organizations. WHCF was using collaborations with other organizations to scale up its impact and take on another city farm site. Likewise, OP was developing partnerships and finding ways to engage with other farmers while leading a network to share their experience related to regenerative farming. Collaborations for mutual learning were also found with research organizations, allowing more inclusive and ‘transdisciplinary’ approaches to knowledge generation. In this way social innovation is a mode of collaborative governance that brings together multiple diverse actors and builds capabilities for co-learning.

#### (ii) Alternative business models and markets for delivery

The second set of social innovation processes are the alternative business models being developed to address the food systems challenges and finance the innovations, thus also enabling new structures and markets to emerge. These can be both social innovations in themselves and ways to scale up other social innovation activities. For example, OP needed to find ways to sell direct to consumers in Leeds and York in order to retain a premium that finances their regenerative approaches. YGA has also created a network of farmers, millers and retail bakers to create their own market from ‘farm to fork’. Members of YGA explained ‘we had no choice but to develop our own market to increase choice to consumers. The mainstream just doesn’t get what we do so we had to develop our own network to get nutritious bread that is environmentally friendly to consumers’.

Other cases were concerned about a reliance on grants and so had developed trading activity to ensure resilience and sustainability of their activities. For example, SA was using a ‘forest school’ business to cover the costs of running its Garden Needs site, while also looking for other pots of funding to support innovations. Sometimes resources are found as part of its contracts with the National Health Service, although there were concerns about the risk of cuts to this public spending. LEYF was able to use surplus from one area of its nursery chain to support other areas in need, but also feared a loss of income and available resources, as funding for nursery places from government was not in line with their rising costs. WHCF combined an income-generating café/hub with public-sector-funded contracts for mental health services. The private cookery classes of Brighton and Hove’s Community Kitchen subsidize its community cookery classes by offering ‘high-end classes with top chefs where people in Brighton Hove pay … and the profit from that goes into running community facing workshops and cookery classes in the same kitchen, so one feeds into the other’ (BH1).

These alternative business models come with their own challenges. In some cases there are not the spaces or opportunities to generate income. For example, BHFP was concerned that its community researcher programme would not continue when the research grant that it is currently part of finishes. Cultivate had also struggled with developing resilient business models and had to close their café as it was not economically viable, especially when operating in a rural area lacking a critical mass of customers.

#### (iii) Engaging with policy and institutional change

For innovation to be genuinely transformational, a key social innovation process is found to be engagement with central and local government as a way to drive social innovation through redirecting strategic investment and policy. The cases show the importance of overcoming challenges related to the ‘silo thinking’ often found in the public sector, and using collaborative approaches to cut across boundaries and bring different voices together. The analysis of the cases also shows how social innovation occurs by acting as a bridge to influence institutional change within specific industries and therefore amplify the learning of the SI. The provision of sustainable food for schools by OP demonstrates that there are opportunities for SIs to act as a lever for change and a demonstration to policy makers of what is possible. LEYF was also found to be influencing the regulation of early years food provision and the development of national guidelines.

Staff in both SA and Cultivate had been asked to conduct research projects for policy makers. This role is particularly important for BHFP, an organization that led the first City Food Strategy in the country by collecting evidence and convening groups to identify alternatives to the current food system. The community researchers are key in this process as they help ensure that local actions and policy developments encompass and speak to local people’s experiences and needs. They therefore receive specific training on writing policy briefings, have been asked to co-deliver workshops, and provide inputs towards the Brighton and Hove Food Strategy.

## 5. Discussion

In answering the first research question (*What are the dimensions and processes of social innovation practice with intent for transforming food systems?*), this study identifies the areas of social innovation practice where there is intent for food systems transformation, although the extent to which these social innovations are transformational can only be assessed over time. In many cases the ‘innovation’ may not be radically novel, but rather incremental or new to a location or context with its specific community needs. The cross-cutting and boundary-spanning nature of food requires an understanding of the breadth of issues, with innovation coming from organizations that may not in themselves be focused on food, but create space for people to engage in discussions and learning about food in ways that traditional public health or environmental education models may not be able to reach. For example, LEYF’s Nursery Chef programme uses its location in an early years provider to bring together a range of ideas to dramatically change how food is integrated into nurseries.

In other cases, the analysis shows potential for more systemic or transformative innovation as they seek structural and institutional change challenging existing relations of power. In most cases, the role of community is key, both as the driver and as the purpose for those innovations to emerge and exist. This is the case with BHFP activities, which organization acknowledged the centrality of communities in social innovations for ‘taking charge of systems that aren’t working’ and ‘tak[ing] it upon themselves to organize things’ (BHFP1).

**Figure 1.**
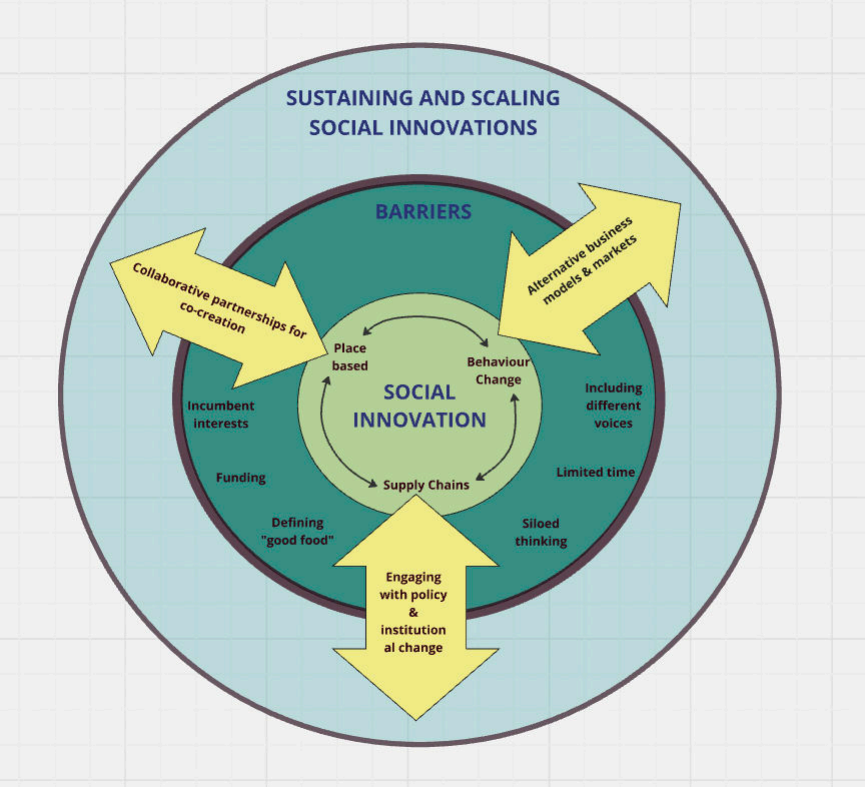
Food system social innovation framework.

The findings can be summarized in a framework for food social innovation with activity found in collaborative place-based services/hubs, supply chain changes addressing power imbalances, and activities for education and behaviour change. Figure 1 also shows the importance of thinking of social innovation as a set of processes including collaborative partnerships for co-creation, the development of alternative business models, and actions for policy/institutional change. In each of these processes, there are important roles for inclusive decision-making, multi-actor networks and open knowledge sharing [24].

In answering the second research question (*What are the challenges to overcome in developing these innovations?*), the analysis also identifies the main challenges facing social innovation for food systems change. As these are unique to their social and geographical context, there is a need to ensure social innovation activities are developing inclusive strategies that build on the diverse (and contested) local understandings of what ‘good food’ means. Overly ‘top-down’ approaches that are perceived to be ‘preaching’ about good food and healthy diets can be alienating. social innovations such as BHFP community researchers and WHCF embed local community voices into alternative approaches to thinking about healthy, sustainable and affordable food.

Further challenges come from ‘incumbent interests’ [30,32], with examples of resistance to change in supply chains where there are a few dominant players. This was found in those social innovations looking to develop local food markets and box schemes, and in innovations aiming to change procurement of food for public sector (such as school food) or larger organizations (such as a chain of nurseries).

Challenges are also found in the lack of capacities and resources for social innovation activities. For some organizations there is a reliance on grants, with the risk of coming to the ‘cliff edge’ of the single large grant. Some case studies addressed this by diversifying their portfolios and having multiple sources of funding, whereas others lacked the capability of seeking and winning multiple grants. Several cases utilized a model of cross-subsidy from their social enterprise trading to provide the finance needed to maintain or scale up their social innovations. However, organizations, particularly those based in rural areas, found that a lack of critical mass of customers reduces trading activity and threatens enterprise viability. Limited resources, time and capacities of smaller enterprises have also been identified as a key issue. For example, BHFP found that farmers were unable to commit to some innovative activity on local supply chains as they needed to prioritize their own survival.

Furthermore, the cost of living crisis has led to growing wage and other bills for the case studies. This issue is exacerbated where funding has not kept up with rising costs. Cost of living was also reported to affect the input and time of volunteers, many of whom had had to take on other work to make ends meet. Special reference was made to the pandemic and the need to address urgent food access, which diverted efforts away from some social innovations. Finally, there are challenges from narrow ‘silo thinking’ in separate government departments that ignores the systemic challenges and leads to a lack of resources for more collaborative research and development.

In relation to our third question (*How can social innovations be amplified and lead to future transformations?*), the analysis and framework show that there are different processes for scaling the social innovation to have more transformative change. Increasing impact through organizational growth was found in some of the cases, with WHCF taking on another city farm site, and SA growing into new areas of well-being services where they are able to get contracts from the public sector. Although such contracts can allow rapid scaling, there may be a need for further support when contracts come to an end and are not renewed.

As well as organizational growth of alternative business models, increased impact can also come about through amplifying and sharing good practice and encouraging others to replicate concepts and models in other places, and by influencing policy makers and other support providers [37]. Scaling is also evident through setting industry standards (e.g. LEYF), as well as having conversations with an extended network of local and national stakeholders. BHFP community researchers expressed their aspirations for community research to be extended to more Food Partnership initiatives and to include more diverse voices and needs.

What is particularly evident is the open sharing of knowledge and replication of social innovations. In this way, social innovation can be distinguished from more commercially oriented innovation as the social aim of the innovation is best met by the sharing of novel ideas, concepts and models rather than protecting them as ‘intellectual property’ (see [39]). This can come about through offering training to other organizations (e.g. LEYF, BHFP), developing toolkits, training volunteers to work in other communities and setting up new initiatives. It also requires collaboration of researchers and practitioners in transdisciplinary approaches, along with a supportive ecosystem involving civil society, private business and government agencies.

## 6. Conclusion

This paper shows how the concept of social innovation can present a useful framework for understanding as well as pursuing food-related social and environmental change. Opening up understandings of innovation is essential in order to include the wider spectrum of practices, services and products that go beyond a technology or a profit-focused logic.

The desire for food systems transformation drives social innovation that breaks down some of the boundaries and silos between social change, sustainability and commercial viability. There is the risk that the lack of a rigid definition can be used to hide a lack of ambition, especially where it is co-opted by incumbent powerful bodies. However, the cases in this paper show that social innovation can occur in small incremental changes that in turn have potential to lead to broader structural and systemic changes in beliefs, values and mindsets for food systems transformation. They also encompass different interacting elements and actors across the food system, creating the space for challenging existing power dynamics in food systems.

The collaborative and boundary-spanning approaches of social innovation require investment in capabilities related to transdisciplinary research that cuts across disciplines and builds relationships between researchers and practitioners [38]. Social innovation also requires conducive policy and institutional contexts that allow these alternatives to flourish, amplify and scale-up. Policy can address the challenges identified in this paper and provide funding for organizations and research projects that aim to address food system transformation.

This paper is limited by its focus on UK qualitative cases. Further research could explore these issues with a wider sample, using quantitative surveys and examining different cultural contexts. However, this paper makes a clear contribution by setting out a framework to help understanding of social innovation related to food systems. In particular, there is potential for future research funding on food systems to ensure social innovation activity is designed into research from the earliest stages.

## Data Availability

The qualitative data supporting this article have been uploaded as part of the online electronic supplementary material [40].

## Footnotes

^1^
For purposes of brevity, abbreviations are used to refer to the organisations that have participated in this research. The full name of each organisation is given the first time the organisations are mentioned in text.
